# Impact of outdoor nature-related activities on gut microbiota, fecal serotonin, and perceived stress in preschool children: the Play&Grow randomized controlled trial

**DOI:** 10.1038/s41598-020-78642-2

**Published:** 2020-12-15

**Authors:** Tanja Sobko, Suisha Liang, Will H. G. Cheng, Hein M. Tun

**Affiliations:** 1 School of Biological Sciences, Faculty of Science, University of Hong Kong, Hong Kong SAR, China; 2 HKU-Pasteur Research Pole, School of Public Health, Li Ka Shing Faculty of Medicine, University of Hong Kong, Hong Kong SAR, China; 3 School of Public Health, Nanjing Medical University, Nanjing, China

**Keywords:** Environmental impact, Microbiome

## Abstract

Due to rapid urbanization, children today have fewer opportunities to interact with nature and this may result in a greater risk for developing stress and depression. Outdoor nature-related activities can enhance general well-being. However, the underlying mechanisms are not fully delineated. Here we recruited 54 preschool children to participate in a 10-week structured nature-related “Play&Grow” program. Following the intervention, children were assessed for connectedness to nature and perceived stress levels using validated questionnaires. Moreover, fecal serotonin level and gut microbiota profiles were measured by ELISA and 16S rDNA amplicon sequencing, respectively. Children were significantly more connected to nature after the intervention. Their gut microbiota altered, especially by modulating the abundance of *Roseburia* and the fecal-serotonin level. Moreover, we also observed a reduction in the overall perceived stress, particularly in the frequency of anger among these children. This study is the first to demonstrate the impact of nature-related activities on gut microbiota, fecal serotonin and psychosocial behaviour of preschool children. However, further mechanistic studies are needed to confirm the functional role of gut microbiota in the association between connectedness to nature and improved psychosocial behavior.

## Introduction

Aggressive behaviour in early childhood, a concerning behavioural problem, has not been taken seriously as a risk factor for violence later in life^1^. A survey of the U.S. population shows that the overall prevalence of inappropriate, intense, or poorly controlled anger is 7.8%^2^. Behavioural problems in children are likewise becoming a prominent issue, particularly, in fast-growing cities like Hong Kong^3^. As a previous study indicated, early intervention is vital as it has been found to lower the risk of developing lifelong mental disorders^4^.

An increased understanding of the brain-gut axis gives evidence to the assertion that the gut microbiome is not only an indicator, but also, a bi-directional influencing factor on mental disorders^5^. Palma’s research found that exposing rat pups to a stressor (i.e., being separated from their mothers) changed their gut microbiota, their stress response, and behaviour^6^. Exposure to the stressor significantly reduced Lactobacilli levels, in particular, a phenomenon also identified in humans during school examinations^7^ and in murine studies utilising prolonged restraint or a short-lasting social stressor^8^. An important neurologically active substance, gamma-aminobutyric acid (GABA), has been found to be produced by the gut microbiome^9^. Relatedly, GABA deficiency is a hallmark of anxiety disorders and major depression^10^. The metabolic by-products of gut microbiota, short-chain fatty acids (SCFAs), have also been found to have a potential contribution to depression phenotype^11,12^ despite more mechanistic information being needed. However, our understanding is still limited in regard to microbiota associated functional changeset in relationship with the mental health of children, despite these indicators.

Another possibly significant link between gut microbiota and behaviours is serotonin, or 5-hydroxytryptamine (5-HT), which is involved in the modulation of a variety of physiological and psychological processes^13,14^. Gut microbiota can both produce and modulate the host’s biosynthesis of serotonin^15,16^. Using germ-free animal models, Yano et al. elegantly demonstrated that microbiota promoted 5-HT biosynthesis from the colonic enterochromaffin cells (EC)^15^, although animal experiments suggest that fecal serotonin can have a pro-inflammatory role and stabilise the gastro-intestinal barrier^17^.

The relationship between intestinal microbiota, brain development, and behaviour has been examined previously^18^, but only a limited number of studies have included exposure to the natural outdoor environment^19^. According to Wilson’s biophilia theory^20^, natural outdoor environments and greenness surrounding environments have been shown to be associated with positive health outcomes^21^, including improved psychological well-being^22^, and decreased the risk of psychiatric disorders^23^, and attention-deficit/hyperactivity disorder symptoms^24^. The increased exposure to natural environments and animals is an important determinant of individual gut microbiome^25,26^, skin microbiome^27^ and salivary microbiome composition^28^. Thus, exposing children to a higher bacterial load in the natural environment by encouraging them to play outside may be a reasonable way to increase the diversity of their intestinal microbiota^19^.

The “Play&Grow” early environmental education programme, with its unique ‘Connectedness to Nature’ component, was designed to increase biophilia and its positive health outcomes for preschoolers. This intervention allows interaction with the natural outdoor world and has proven to be effective in encouraging healthy lifestyle behaviours in families with preschoolers in prior experiments^29,30^. Moreover, the intervention increases the vegetable consumption of children^31^. The main objective of this trial was therefore to investigate the impact of the “Play&Grow” intervention on the intestinal microbiome (both taxa and predicted functions), gut serotonin level, and the psychological well-being of 2–5 years old children.

## Results

**Figure 1:**
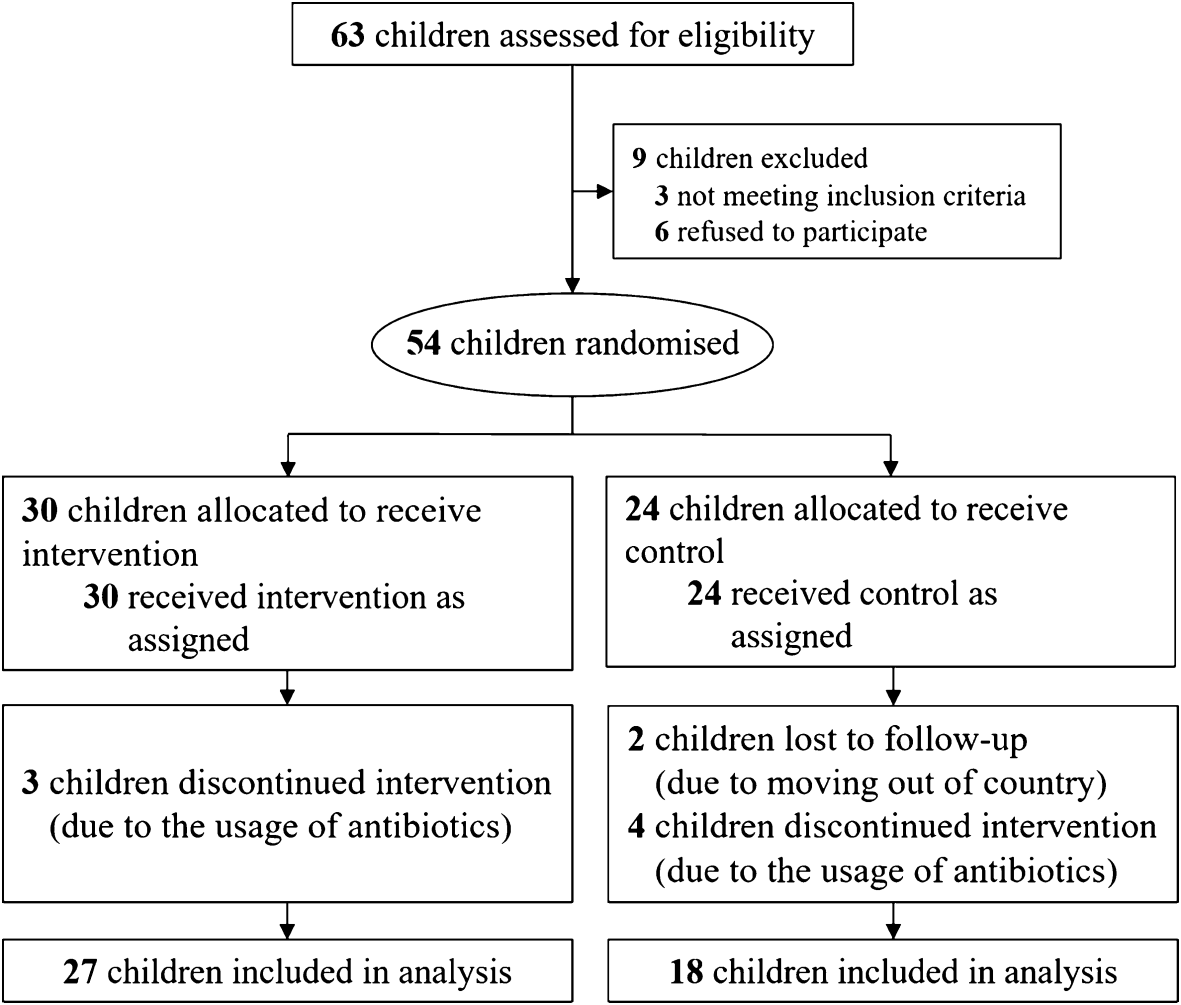
Flowchart of the RCT.

In total, fifty-four participant families were randomly assigned to the intervention (IG) (*n* = 30) or control groups (CG) (*n* = 24). Forty-five participants completed the pre- and post-intervention questionnaires and 84% of them provided the fecal samples (*n* = 27 in the IG and *n* = 18 in the CG) (Fig. 1). The demographics of the two groups are listed in Supplementary Table 1. Comparisons on demographics between study groups and their respective dropouts revealed no significant difference at baseline, indicating no impact of the dropout on the results of the study (Supplementary Table 1a and 1b).

### Increase in connectedness to nature

The 10-week intervention resulted in increased connectedness to nature (CN) within the IG children. The total CN score in the IG increased significantly post-intervention (*p* = 0.04, 3.54 vs. 3.85), compared to the CG (*p* = 0.20, 4.03 vs. 4.04) (Supplementary Fig. 1a). Specifically, the scores for one of the factors, “Responsibility towards Nature” (RN) significantly increased from pre- to post-intervention (*p* = 0.003, 3.33–3.67) (Supplementary Fig. 1b).

### Fecal serotonin & improvement of psychosocial behaviour

**Figure 2:**
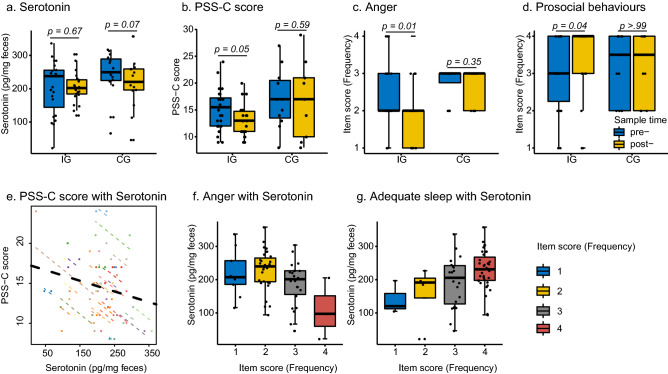
Change in serotonin and child psychosocial measurements. (**a**) Change in fecal serotonin between IG (n = 23) and CG (n = 15), pre- and post-intervention. (**b**) Change in PSS-C score in child psychosocial measurements between IG (n = 20) and CG (n = 9). (**c**) Change in score of anger in child psychosocial measurements between IG (n = 24) and CG (n = 12). (**d**) Change in score of prosocial in child psychosocial measurements between IG (n = 24) and CG (n = 12). (**e**) Association between serotonin and PSS-C score. (**f**) Association between serotonin and anger frequency. (**g**) Association between serotonin and adequate sleep. In (**a**) to (**d**) blue colour indicated pre-samples, yellow colour indicated post-samples. In (**e**), different colour of node and dotted-line indicated different individual, and the black dotted-line indicated the overall association. In (**f**) and (**g**), four colour indicated four degrees of anger and adequate sleep frequency.

A stable level of fecal serotonin was observed in the IG while a decreasing trend was found in the CG (*p* = 0.07, 249.36–220.60 pg/mg of feces) (Fig. 2a). Overall score related to the perceived Stress Scale for Children (PSS-C) and specific scores of anger frequency and prosocial behaviour improved significantly post-intervention (*p* = 0.05, *p* = 0.01 and 0.04, respectively, Fig. 2b–d) compared with the control group. We further separated the participants into three groups according to the changes in their PSS-C scores between pre- and post-intervention. A total of 65% of participants in IG (n = 13) have decreased PSS-C scores compared with 22% in the CG group (n = 2, Fisher's Exact Test p = 0.01). Especially, three children with the highest anger score improved (from level 4 to level 2) following the intervention. In addition, three other children who had higher anger scores of level 3 also decreased to level 2. Following the intervention, the two children with the lowest prosocial behaviour scores improved (Fig. 2d). Fecal serotonin was found to have a negative correlation with the overall PSS-C score (Repeated measures correlation, r = −0.45, *p* value = 0.02, Fig. 2e). It was also found to have an increasing trend of association with sleep frequency (Jonckheere-Terpstra test: JT = 832, *p* value < 0.01), and have a decreasing trend with anger frequency (Jonckheere-Terpstra test: JT = 475.5, *p* value = 0.01) (Fig. 2f–g). The relationship of fecal serotonin with the frequency of anger was independent to the age of the children (Supplementary Table 2).

### Changes in the gut microbiome

**Figure 3:**
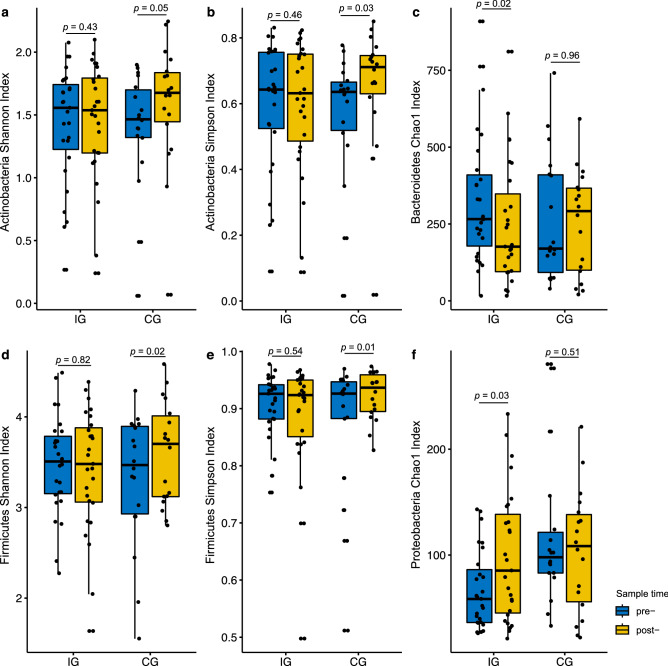
Alpha diversity changes between pre- and post-intervention in IG (n = 27) and CG (n = 18). (**a**) Shannon index of Actinobacteria. (**b**) Simpson index of Actinobacteria. (**c**) Chao1 index of Bacteroidetes. (**d**) Shannon index of Firmicutes. (**e**) Simpson index of Firmicutes. (**f**) Chao1 index of Proteobacteria. Blue colour indicated pre-samples, yellow colour indicated post-samples.

An average of 49,577 quality-filtered reads (per sample) were generated (Supplementary Table 3) and a total of 14 phylum, 176 genus, and 255 species were detected in gut microbiomes of the participants. The most abundant phyla of gut microbiota were Firmicutes, Bacteroidetes, and Proteobacteria (Median abundance: 0.44, 0.27, and 0.11%) (Supplementary Fig. 2). Although no notable difference in the overall alpha diversity between two time points both in IG and CG groups, the participants from the IG with decreased PSS-C score exhibited a significantly higher gut microbiota richness. (Supplementary Fig. 3). The richness of Bacteroidetes and Proteobacteria phyla changed significantly in the IG after the intervention. The Chao1 richness of Bacteroidetes significantly decreased (*p* < 0.01), while that of Proteobacteria increased (*p* = 0.03). On the other hand, there were no significant changes in the diversity of Actinobacteria and Firmicutes (both Shannon and Simpson indices) in the IG compared with the increased diversity in the CG following the intervention (Fig. 3).

**Figure 4:**
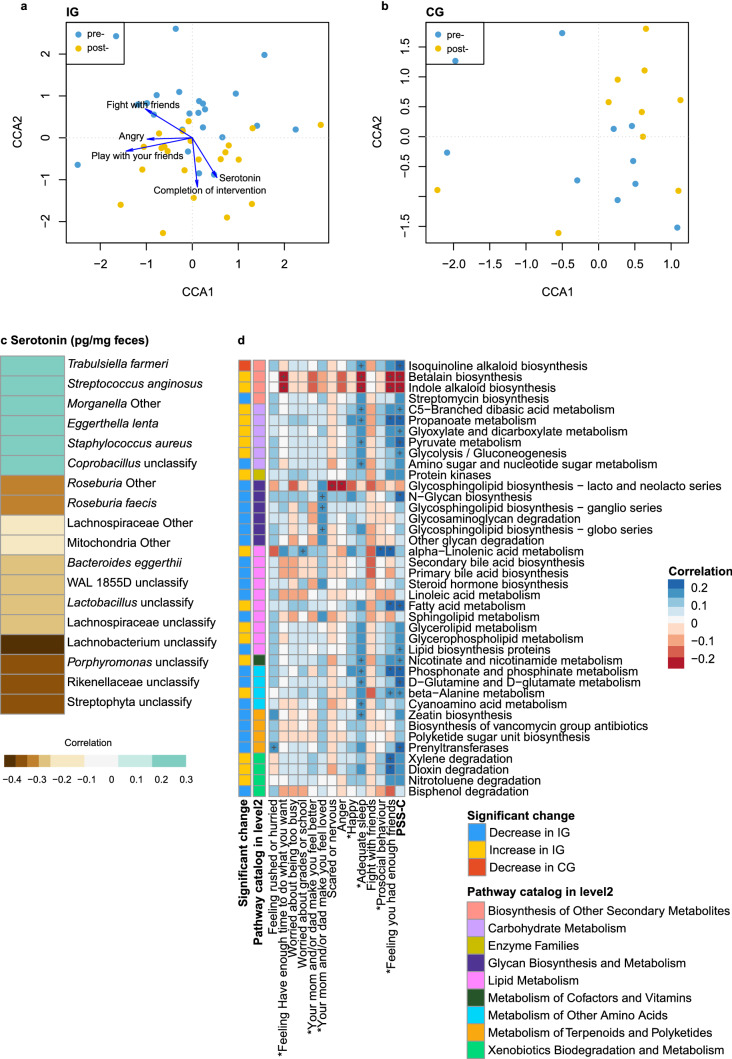
Associations between serotonin, stress items and gut microbiome. (**a**) and (**b**) Canonical Correlation Analysis (CCA) in CG and IG samples. Blue node indicated the pre-intervention samples; yellow node indicated the post- samples. (**c**) Heatmap of correlations between serotonin and gut microbiota species. Only the significant associated factors on gut microbiota in spearman correlation were listed. Colour in each cell of the heatmap indicated spearman correlation of serotonin and species abundance. Only the *p* value of association less than 0.05 were chosen for display in this figure. (**d**) Associations between gut microbiota functions and stress items, and changes in microbiota functions by the intervention. The heatmap indicated the spearman correlation between metabolic functions of gut microbiota and children’s stress items. +*p* value less than 0.1; *p value less than 0.05; **p value less than 0.01. *in the front of the column name indicated we used the reversed score rather than the original score.

No significant difference in microbial community structure was observed between the IG and the CG at baseline; however, a clear difference between pre- and post-measurements in both IG and CG groups was observed based on the Canonical Correspondence Analysis (CCA) results. Several factors including completion of the intervention, serotonin levels, and anger frequency, were identified to influence the gut microbiota of children in the IG but not in the CG (Fig. 4a, b). Changes in certain species were found post-intervention (Supplementary Fig. 4). Interestingly, an increased abundance of *Roseburia* was shown in the CG but not in the IG (Supplementary Fig. 4g). The abundance of an unknown species of *Bacteroides, Parabacteroides distasonis,* an unclassified *Acidaminococcus*, an unclassified *Dialister*, and an unclassified *Bilophila* decreased in the IG, while an unclassified species of *Blautia* increased in the IG*.*

### Correlation between bacterial, serotonin and psychosocial behaviours

Among the microbiota that were significantly correlated with fecal serotonin levels, six of the species showed positive correlation, while the other twelve correlated negatively (Fig. 4c). Although statistically not significant, there was a negative trend of correlation between fecal serotonin level and Shannon diversity index of Bacteroidetes (*p* = 0.07, ρ = −0.21) and Chao1 richness index of Firmicutes (*p* = 0.09, ρ = −0.19), but a positive correlation with Proteobacteria richness (*p* = 0.06, ρ = 0.22) (Supplementary Fig. 5).

**Figure 5:**
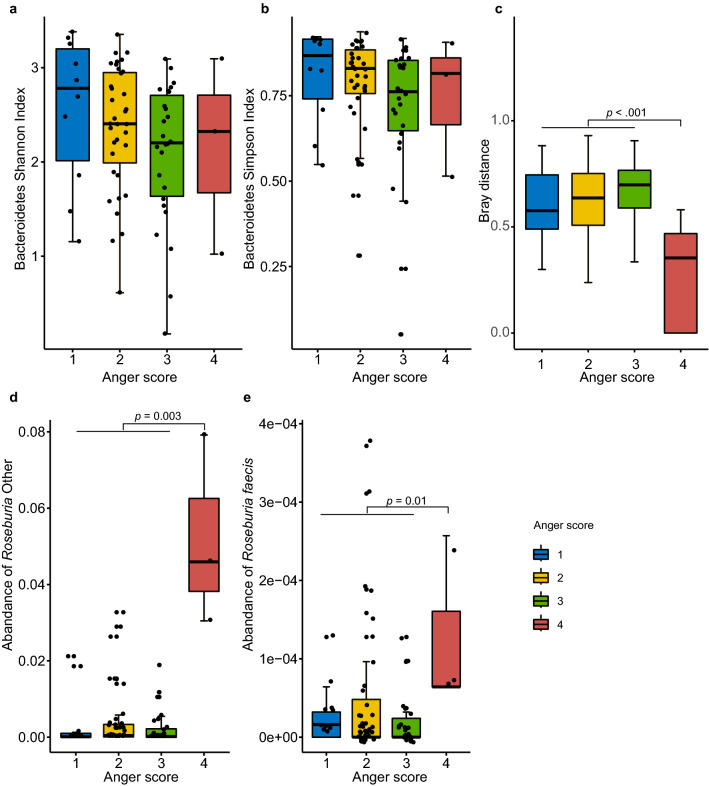
Associations between gut microbiota and Anger frequency. (**a**) Association between Shannon index of Bacteroidetes and anger frequency. (**b**) Association between Simpson index of Bacteroidetes and anger frequency. (**c**) Association between bray distance of species level and anger frequency. (**d**) Association between the abundance of *Roseburia Other* species and anger frequency. (**e**) Association between the abundance of *Roseburia faecis* species and anger frequency. Four colours indicated four degrees of anger frequency.

Importantly, an unknown species of *Roseburia,* which increased in the CG post-intervention, was significantly negatively correlated with fecal serotonin levels (*p* = 0.01, ρ = −0.31) (Fig. 4c), as was another species under the same genus, *Roseburia faecis* (*p* = 0.01, ρ = −0.31). The association between serotonin and *Roseburia* could be future conformed by the logistic regression (Supplementary Table 4). Moreover, *Trabulsiella farmeri, Streptococcus anginosus, Eggerthella lenta*, and *Staphylococcus aureus* showed positive correlations.

The overall PSS-C score was negatively correlated with Chao1 richness (*p* = 0.03, ρ = −0.27, Supplementary Fig. 6a), Shannon diversity (*p* = 0.03, ρ = −0.26, Supplementary Fig. 6b), and Simpson diversity (*p* = 0.05, ρ = −0.24, Supplementary Fig. 6b–c) of Bacteroidetes. A similar trend was observed for Shannon diversity of Proteobacteria (*p* = 0.07, ρ = −0.23, Supplementary Fig. 6d) although statistically not significant. Besides, Shannon and Simpson diversity of Bacteroidetes showed a decreasing trend according to anger frequency (Jonckheere-Terpstra test: JT = 674, *p* value = 0.02; JT = 666, *p* value = 0.02, Fig. 5a, b). Although no notable difference was detected in overall alpha diversity, children with the highest anger frequency shared more similar gut microbial community when compared to children with fewer anger problems (Fig. 5c). On a species level, two species of *Roseburia* were significantly higher in the microbiome of children who had the highest anger frequency (Fig. 5d, e).

### Alteration in predicted microbiota functions

A total of 273 microbiota functional categories (at KEGG level-3, www.kegg.jp/kegg/kegg1.html) were predicted using PICRUSt. Under the functional category of “Metabolism”, 125 metabolic functions were detected with the highest abundance in “Purine metabolism.” Among those metabolic functions, we observed more functional changes in the IG (*p* < 0.05), in which 22 pathways decreased and 17 pathways increased. Comparatively, only a single pathway decreased (Supplementary Fig. 5) in the control group. At KEGG level-2, a significant correlation was observed between the overall PSS-C score and various amino acid metabolisms covering Phosphonate and phosphinate, d-Glutamine and d-glutamate, and beta-Alanine. Furthermore, the overall PSS-C score was positively correlated with the metabolism of carbohydrates including C5-Branched dibasic acid, Propanoate, Glyoxylate and dicarboxylate, Pyruvate, and Glycolysis/Gluconeogenesis. Betalain and Indole alkaloid biosynthesis, which have been increased by intervention, was negatively correlated with the total PSS-C score as well as the other sub-scores of the PSS-C questionnaire (Fig. 4d). However, none of these altered pathways showed a significant correlation to the fecal serotonin level in general.

## Discussion

To investigate the impact of Connectedness to Nature (CN), we conducted a randomized trial and subsequently studied its impact on changes in gut microbiota and serotonin as well as the psychosocial behaviour of children. In this study, we observed that early environmental education intervention significantly reduced the overall perceived stress, particularly anger frequency among preschool children, modulated the abundance of certain gut microbiota, and in contrast to the respective control condition, did not reduce gut serotonin levels. The “Play&Grow” programme was successful in modulating the microbiome within the IG after regular exposure to nature over the course of 2 months. Similarly, a recent study in an adult population demonstrated that dipping hands in soil daily for 2 weeks changed the skin and gut microbiomes of the study’s participants, indicating a correlation between natural interactions and the diversity of the gut microbiota^32^. In particular, our study discovered that exposure to natural bacteria moderated the diversity of the Actinobacteria and Firmicutes phyla, decreased the alpha diversity of the Bacteroidetes phylum, and increased the alpha diversity of Proteobacteria. We also observed inter-individual variation in changing gut microbiota diversity due to the intervention, and it might be related to the variation of exposure intensity among individuals.

Velles-Colomer et al. has recently reported an important association between depression, lower quality of life, and *Bacteroides* in the large Flemish Gut Flora Project^33^. Our controlled intervention allows us to suggest that exposure to bacteria from natural environmental could be beneficial to behavioural outcomes; however, this intriguing possibility needs further investigation. According to the natural history of gut microbiome development in early-life, the variation in the abundance of *Bacteroides* species among individuals is large due to different exposure levels and other factors associated with early life (e.g. birth mode and breastfeeding)^34,35^. Thus, the impact of our intervention on these particular taxa might be hindered by various factors. Despite this, we observed less individual variation of *Bacteroides* taxa following the intervention, which implies a movement toward modulation of the gut microbiome could be related to increased exposure to the natural environment and connectedness to nature^34,36^. During the first years of life, exposure to the natural microbial experience is therefore an important moderator for normal developmental patterns of gut microbiota and behaviour.

The current study showed positive changes in the psychosocial scores of participants in the IG after the “Play&Grow” intervention. This is in agreement with the systematic review of studies that found a beneficial impact of exposure to the natural environment on negative emotions (i.e., anger and sadness)^37,38^. Moreover, living in close proximity to forests is known to have salutogenic effects on the amygdala^39^. Likewise, the “Play&Grow” programme significantly improved the overall perceived stress, particularly anger frequency and prosocial behaviour of participants, which suggests a link between these behaviours and the bacterial load encountered by the participants. This is a new area of research, lacking the human data (although preclinical studies are promising); comparison of our results with other studies on similar age groups is therefore difficult. Studies in germ-free (GF) animals showed that GF mice were significantly socially impaired, yet this deficit could be repaired following bacterial colonisation, suggesting that gut microbiota affects psychosocial behaviour^40^.

Identification of the bacteria that is involved in these new mechanisms may help to understand the nature of the psychosocial consequences of bacterial alterations early in life. Our intervention decreased the prevalence of *Parabacteroides distasonis*, which has been positively associated with exposure to social stressors in mice^41^. A lower abundance of *Roseburia* has been reported in schizophrenics, but the opposite trend was found in people with mood disorders^42,43^. In this study, we also found that our intervention somewhat maintained *Roseburia*, whose abundance increased over time in the CG. Our results suggest that exposure to bacteria in nature may control the growth of *Roseburia*, resulting in a stabilization of gut serotonin levels. This indicates that the changes to the gut microbiota following exposure to natural environments may influence behaviours of preschool children. The gut microbiota related serotonin (5HT) pathway has already been proposed in animal models; but it has not yet been well-studied in human subjects, especially in children.

The “Play&Grow” programme’s components align with biophilia theory. Both structured and unstructured nature experiences (i.e., playing with leaves and soil) and educational messaging (i.e., “it is ok to get your hands dirty with soil”) increased children’s contact with the natural environment and associated health outcomes. It strengthened the idea that nature is a “playground” and provided an opportunity for highly-urbanized children to reconnect to nature and interact with the microbiome of the natural environment.

Our intervention may be seen as an extension of the “Hygiene Hypothesis” also known as the “Microbiota Hypothesis”, which suggests that exposure to bacteria and other pathogens may actually be beneficial to the education and development of the immune system^44–49^. Studies have reported that reduced exposure to microbes in modern urban societies may increase vulnerability to neurodevelopmental disorders (including autism spectrum disorders (ASD) and schizophrenia) and stress-related psychiatric disorders (including anxiety and mood disorders)^46,50–53^. Our study also showed this association, indicating children with the highest anger frequency had the least inter-individual variation in gut microbial community and the highest levels of *Roseburia* species.

This study investigated a possible relationship between fecal serotonin, the gut microbiome, and psychosocial behaviours. While a trend towards decreased fecal serotonin level was observed only in the CG, serotonin levels stayed stable in the IG. Moreover, serotonin is negatively correlated with the diversity of Bacteroidetes, Firmicutes, and Proteobacteria, as well as with the abundance of *Roseburia.* A recent study identified *Roseburia* as potential serotonin producers; however, the degradation pathway of serotonin by the bacteria is still not yet fully elucidated^33^. Hence, our study indicates that the Play&Grow intervention in preschool children increased connectedness to nature, improved children’s moods, especially with respect to their anger levels, as well as changed the gut microbiome (especially the abundance of *Roseburia*), and in contrast to the respective control group, did not decrease gut serotonin levels. But due to the limitation of database and software, we didn’t find the significant difference related to serotonin metabolism, so the associations need to be further confirmed by more targeted studies.

Besides the changes observed in microbial taxa, we also identified that our intervention significantly altered the microbiota functions within participants of the IG when compared with those in the CG. Among the functional changes, the most noteworthy one was a rise in Indole alkaloid biosynthesis, which also has a negative correlation with the PSS-C score, has been previously reported as a potential drug for depression and anxiety^54^. Another remarkable functional change was the decreased abundance of metabolic functions related to the biosynthesis of glycosphingolipid. A previous study also reported an increase in glycosphingolipid biosynthesis in Chinese children with autism spectrum disorders^55^, indicating a potential disadvantage to an abundance of glycosphingolipid. In general, the functional role of glycosphingolipids in the central nervous system have been detected^56^, but mechanistic study is needed to explore their roles in the behavioural development of children.

This is a pioneer study that links a nature-related intervention with psychosocial outcomes and modulating gut-microbiota-associated serotonin levels, suggesting a potential pathway to highlight the benefit of connectedness to nature. While our data are encouraging, certain limitations should be mentioned. Although there is a diurnal fluctuation of serotonin levels in the blood, this is still unobserved in fecal samples. Our study did not take account of the fecal sample collection time in order to have more flexibility for participants. Moreover, the sample size and the duration of the intervention may have significantly influenced the results of our study. Due to the limitation of the 16S rRNA reference database, our study could not provide species-level resolution for some taxa. Further studies with a larger sample size coupled with metagenomic sequencing are warranted. Despite these limitations, the findings of this pilot trial might be important in contributing to further related investigations.

## Conclusion

In our early environmental educational intervention study, we demonstrated the impact of nature-related activities on gut microbiota, fecal serotonin, and in improving psychosocial behaviour of preschool children. The impact included the changes in the diversity of microbiota, modulation of *Roseburia* abundance, prevention of a downregulation of fecal serotonin levels, and the improvement of psychosocial behaviours of children. Our findings suggest that the gut microbiota can be a target for further studies on behavioural modifications and mental health interventions in a wider perspective. Further mechanistic studies are needed to confirm the mechanistic contribution of gut microbiota into the association between connectedness to nature and improved psychosocial behaviour.

## Material and methods

### Informed consent and ethics

This study was approved by the human research ethics committee (HREC) of Hong Kong University, and written informed consent was obtained from all of the participants’ parents or legal guardians and the participants themselves. Trial registration: ClinicalTrials.gov, NCT02715544. Registered 22 March 2016. All methods were performed in accordance with the relevant guidelines and regulations by HREC.

### Intervention design

A two-arm, randomized controlled trial (RCT) with masked outcome assessment—“Play&Grow”—was developed as a family-oriented, early environmental education programme for families with preschool children at the University of Hong Kong^29,30^. Primarily, the “Play&Grow” programme aims to reconnect preschoolers to nature and induce changes in health behaviours and outcomes by having outdoor activities that promote exposure to nature. On previous studies and calculation principles and methods^57^, the sample size (α = 0.05, power of 0.8) needed to detect the effect of the “Play&Grow” intervention on other related outcomes was 100 families in each group of RCT (including an allowance for 20% attrition). However, the sample size of this proof-of-principle study was not determined by a formal power calculation given the exploratory nature of the study. The assumption was that at least 30 children in total would be recruited from two groups: intervention group (IG) or Control group (CG). They were measured twice: before and after the intervention and the samples analysed for gut microbiota.

### Participants

Children, aged two to five years, were recruited to participate in this programme, together with their main caregivers, via online advertisement at the beginning of 2018. The exclusion criteria were children from non-local families, children who were on antibiotic therapy in the two months prior to the start of the programme, and children with chronic health conditions.

### Procedures

Consenting families (*n* = 54) were randomly assigned to the Intervention (IG)(*n* = 30) or Control Groups (CG)(*n* = 24) using a random computer-generated number from 0 to 1 before the start of the trial, which was in April-June 2018. Participants were assigned to the IG if their number was > 0.5, and to the CG if their number was < 0.5. During the randomization process, no stratification or blocking were performed. All participants completed the pre- and post-intervention questionnaires, and 84% provided fecal samples (*n* = 27 in the IG (3 lost to antibiotic usage during the study, or failure to complete the study) and *n* = 18 in the CG (6 lost to antibiotic usage during the study, a failure to complete the study, or were lost to follow up) (Fig. 1). The demographics of the two groups are listed in Supplementary Table 1. The study aims and hypothesis were not discussed with any participants; they were only informed that they would be participating in a family health promotion programme. All measurements were done pre- and post-study on both groups, and all related research assistants, the questionnaire typist, and study statisticians were not informed of the group allocations, measurements and outcomes.

#### Intervention group (IG)

The intervention was held once per week for ten consecutive weeks, from June 2018, in public parks throughout the Hong Kong SAR. Topics and elements of the programme were developed prior to the start of the intervention and were discussed in detail^29^. Each session, led by a pair of research assistants, included a guided nature activity that promoted “hands-on” experiences with materials found in nature. These activities were specifically designed for IG participants to provide them an opportunity to come into contact with the microbiome of the natural environment. Some additional “homework”, such as collecting nature subjects, making nature art, and growing plants, was given to the families in the IG to further increase their interaction with the outdoor environment and its bacteria. A package of healthy lifestyle recommendations published by the Hong Kong government did not have any special dietary change recommendations and was available to all caregivers in Hong Kong^58^.

#### Control group (CG)

In order to eliminate potential confound factors, i.e. changes in gut microbial composition as a result of any dietary changes, both groups were reminded of the resource mentioned above^58^.

### Harms

This study focused on exposing children to the nature environment and did not contain any clinical procedure/intervention. As a result, no considerable harm or unintended effect were noted during the trial.

### Outcome measurement

All measurements were conducted prior to and after the 10-week intervention. Detailed procedures for children’s psychosocial measurement, assessment for connectedness to nature (CN), serotonin measurement, and gut microbiota analysis were indicated in the Supplemental material.

### Statistical analysis

With the recommended pipeline in QIIME, the relative abundance of OTUs was summarised at phylum, genus and species level. Microbial alpha diversity was calculated by Chao1, Shannon, and Simpson indices. The differences between two time-points within groups were identified by the Wilcoxon signed-rank test (R package stats), including the difference of CN factor scores, microbiota diversity based on OTU, and species level, microbiota alpha diversity for each of the phylum, serotonin, and stress levels. Jonckheere-Terpstra test was used to test the trend (R package clinfun). The association between microbiota diversity, species, and stress levels with serotonin levels was measured by Spearman's rank correlation and logistic regression (R package stats) by category the continue number by the median. The association between PSS-C score with serotonin levels was measured by repeated measures correlation coefficient (R package rmcorr). CCA was performed based on species profile of the IG and the CG, separately (R package vegan).

## Supplementary Information

Supplementary Information.

## Data Availability

The sequence data under this study are publicly available in the European Nucleotide Archive under accession number PRJEB34058. Dr. Tun had full access to all subjects’ meta-data and associated microbiota data in the study and takes responsibility of correspondence for data requests related to this study.
