# Supplementary material for: Impact of outdoor nature-related activities on gut microbiota, fecal serotonin, and perceived stress in preschool children: the Play&Grow randomized controlled trial
Source: Sci Rep. 2020 Dec 15;10:21993. doi: 10.1038/s41598-020-78642-2 (PMC7738543; doi:10.1038/s41598-020-78642-2)
Supplement: Supplementary file 1 — Supplementary Information. [file 41598_2020_78642_MOESM1_ESM.docx]

**Impact of outdoor nature-related activities on gut microbiota, fecal serotonin, and perceived stress in preschool children: the Play&Grow randomized controlled trial**

**Tanja Sobko**^1,+^, **Suisha Liang**^2,+^, **Will H.G. Cheng**^1^**, Hein M. Tun**^2,3,*^

^1^School of Biological Sciences, Faculty of Science, University of Hong Kong, Hong Kong SAR

^2^HKU-Pasteur Research Pole, School of Public Health, Li Ka Shing Faculty of Medicine, University of Hong Kong, Hong Kong SAR

^3^School of Public Health, Nanjing Medical University, Nanjing, China

^+^These authors contributed equally in this manuscript.

*Corresponding author heinmtun@hku.hk

**Supplementary Methods**

**Primary outcomes and their assessment**

All measurements were conducted prior to and after the 10-week intervention.

**Children’s psychosocial measurement -** **Children’s stress questionnaire**

The 14-item perceived stress questionnaire about stress related to home and school, behaviour, and health in children^1^ was filled-out jointly by children and their parents. The items were rated on a four-point Likert-scales, from 1 point (never) to 4 points (a lot). Perceived Stress Scale for Children (PSS-C) score indicated by the sum of score of 2-14 question (Questions 3, 6, 7, 10, 11, 13 and 14 are reversed scored).

Similar to the adult Perceived Stress Scale, which also focuses on chronic stress perception^2^, PSS-C is a recently developed US-based screening tool that aimed to assist researchers and clinicians to identify children with stress/anxiety disorder^1^. In White’s study, the utility and validity of PSS-C on its ability to discriminate such has been reported^1^. To our knowledge, some latest studies have been utilizing PSS-C as a mean to validate other scales^3 4^.

**Connectedness to Nature (CN)**

“Connectedness to Nature” in children was measured using the validated CNI-PPC tool (16 items, 4 factors) ^5^. The items were rated on a five-point Likert-scale, from 1 point (never) to 5 points (always). The extent of CN was evaluated using the total CN score and the mean points of each of the four factors: enjoyment of nature, empathy for nature, responsibility towards nature, awareness of nature.

**Serotonin measurement**

The content of serotonin was determined by using the Serotonin Ultrasensitive ELISA kit according to the manufacture’s protocol (Eagle Biosciences, Amherst, NH, US). 0.2 g of fecal samples were homogenised with 1 mL phosphate-buffered saline (PBS) containing 0.1% ascorbic acid. Samples were further diluted with PBS. The standard curve was constructed from the standards provided in the ELISA kit. A four-parameter logistic curve was set up with a standard at the following concentrations: 0, 0.67, 2, 6.7, 20 and 100 pg/sample.

**Gut microbiota analysis**

Fecal samples of preschool children were collected before and after the intervention by a standard protocol. Total DNA from fecal was extracted using QIAamp PowerFecal DNA Kit (Qiagen, Venlo, the Netherlands). The concentration and quality of DNA were assessed using nanodrop and agarose gel electrophoresis, respectively. The PCR-based amplicon library targeting the V3-V4 variable region of the 16S rRNA gene was performed using universal bacterial primers: 341F:5’ ACT CCT ACG GGA GGC AGC AG 3’, 806R:5’ GGA CTA CHV GGG TWT CTA AT 3’, and then sequenced on an Illumina Miseq platform at BGI (Shenzhen, China) using 300 bp paired-end (PE) sequencing. Bioinformatic analysis was performed using the QIIME pipeline ^6^. Forward and reverse reads were merged using Flash followed by removing the chimera sequence by mapping the merged reads to the GREENGENES database. Operational taxonomic units (OTUs) were picked and quantified using the open-reference OTU picking protocol by searching reads against the GREENGENES database. To avoid bias due to variation in sequencing depths among samples, data were rarefied to 31,114 sequences per sample. The PICRUSt pipeline^7^ (version 1.1.3) was used to predict microbiota functions. Briefly, the closed reference OTUs were annotated using 16S rRNA gene sequences and followed by normalisation based on kwon/predicted 16S copy numbers of different taxa. Then, the functional traits of microbiota were predicted and summarised into three different levels of KEGG Orthologs (www.kegg.jp/kegg/kegg1.html).

**References**

1. White BP. The Perceived Stress Scale for Children: A Pilot Study in a Sample of 153 Children. *International Journal of Pediatrics and Child Health* 2014;2(2):45-52.

2. Cohen S, Kamarck T, Mermelstein R. Perceived stress scale. *Measuring stress: A guide for health and social scientists* 1994;10:1-2.

3. Soheili F, Hosseinian S, Abdollahi A. Development and Initial Validation of the Children’s Hardiness Scale. *Psychological reports* 2020:3329412094517. doi: 10.1177/0033294120945175

4. Gonener A. Effect of Sport Education on Stress and Assertiveness of Middle School Students. *International education studies* 2020;13(5):108. doi: 10.5539/ies.v13n5p108

5. Sobko T, Jia Z, Brown G. Measuring connectedness to nature in preschool children in an urban setting and its relation to psychological functioning. *PLoS ONE* 2018;13(11):e0207057. doi: 10.1371/journal.pone.0207057

6. Caporaso JG, Kuczynski J, Stombaugh J, et al. QIIME allows analysis of high-throughput community sequencing data. *Nat Methods* 2010;7(5):335-6. doi: 10.1038/nmeth.f.303 [published Online First: 2010/04/13]

7. Langille MG, Zaneveld J, Caporaso JG, et al. Predictive functional profiling of microbial communities using 16S rRNA marker gene sequences. *Nat Biotechnol* 2013;31(9):814-21. doi: 10.1038/nbt.2676 [published Online First: 2013/08/27]

**Supplementary Figures**


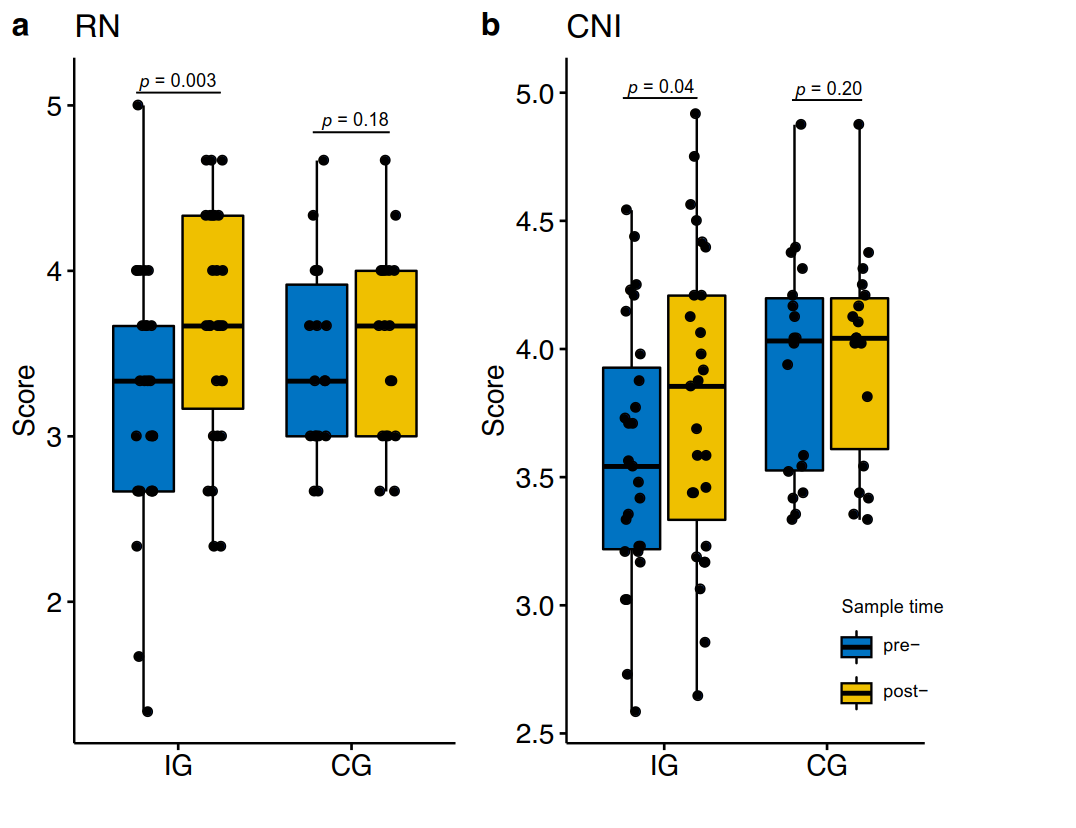


**Supplementary Figure 1. Change in children’s connectedness to nature score in IG (n=27) and CG (n=18).**

a, Responsibility towards Nature (RN) score. b, Total Connectedness to Nature (CN) score. Blue colour indicated pre- samples, yellow colour indicated post- samples.


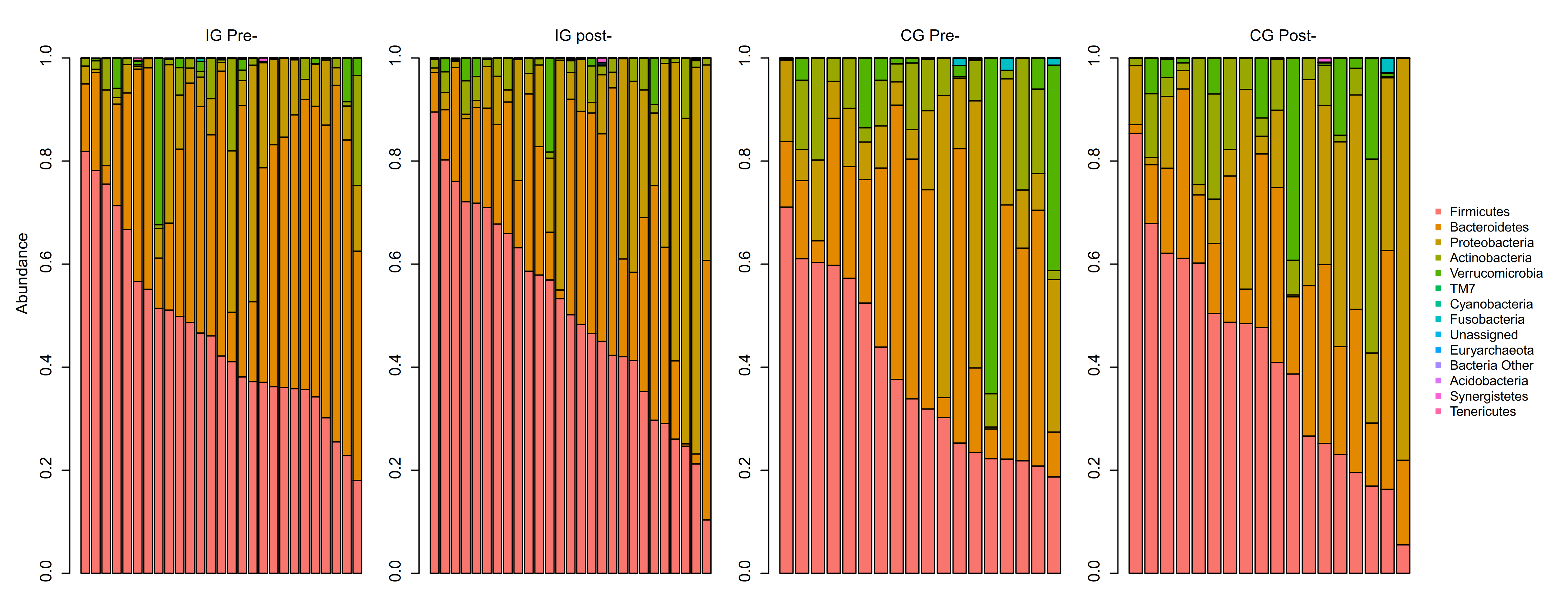


**Supplementary Figure 2. Bar plot of phylum level in children fecal samples.**

All phyla in children fecal samples. Fecal samples were grouped by pre- and post-intervention or control.


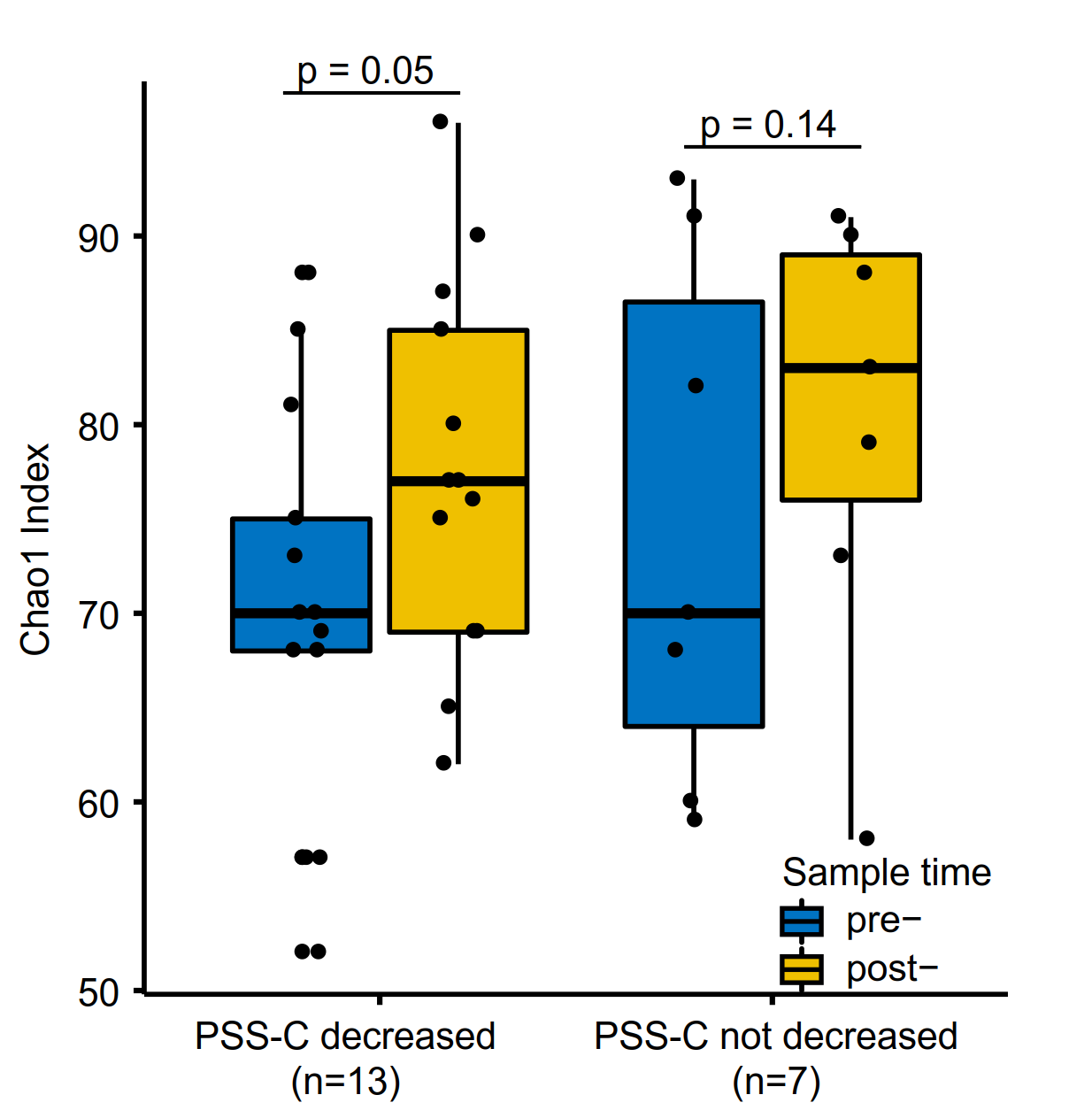


**Supplementary Figure 3. Change in Chao1 index in PSS-C score decreased or not-decreased sample.**

The participant in IG group were separated into 2 groups: PSS-C score decreased or not, depending on whether they have a decrease score after the intervention. Blue colour indicated pre-intervention samples, yellow colour indicated post-intervention samples.

**Supplementary Figure 4. Species abundance changes between pre- and post- internveiton (n=27) or control (n=18) samples.**

a, *Bacteroides Other*. b, *Parabacteroides distasonis.* c, *Clostridiales unclassify*. d, *Clostridiaceae unclassify*. e, *Pseudoramibacter Eubacterium unclassify*. f, *Blautia unclassify*. g, *Roseburia Other*. h, *Acidaminococcus unclassify*. i, *Dialister unclassify*. j, *Erysipelotrichaceae unclassify.* k, *Bilophila unclassify*. l, *Enterobacteriaceae Other*. Blue colour indicated pre-intervention/control samples, yellow colour indicated post-intervention/control samples.


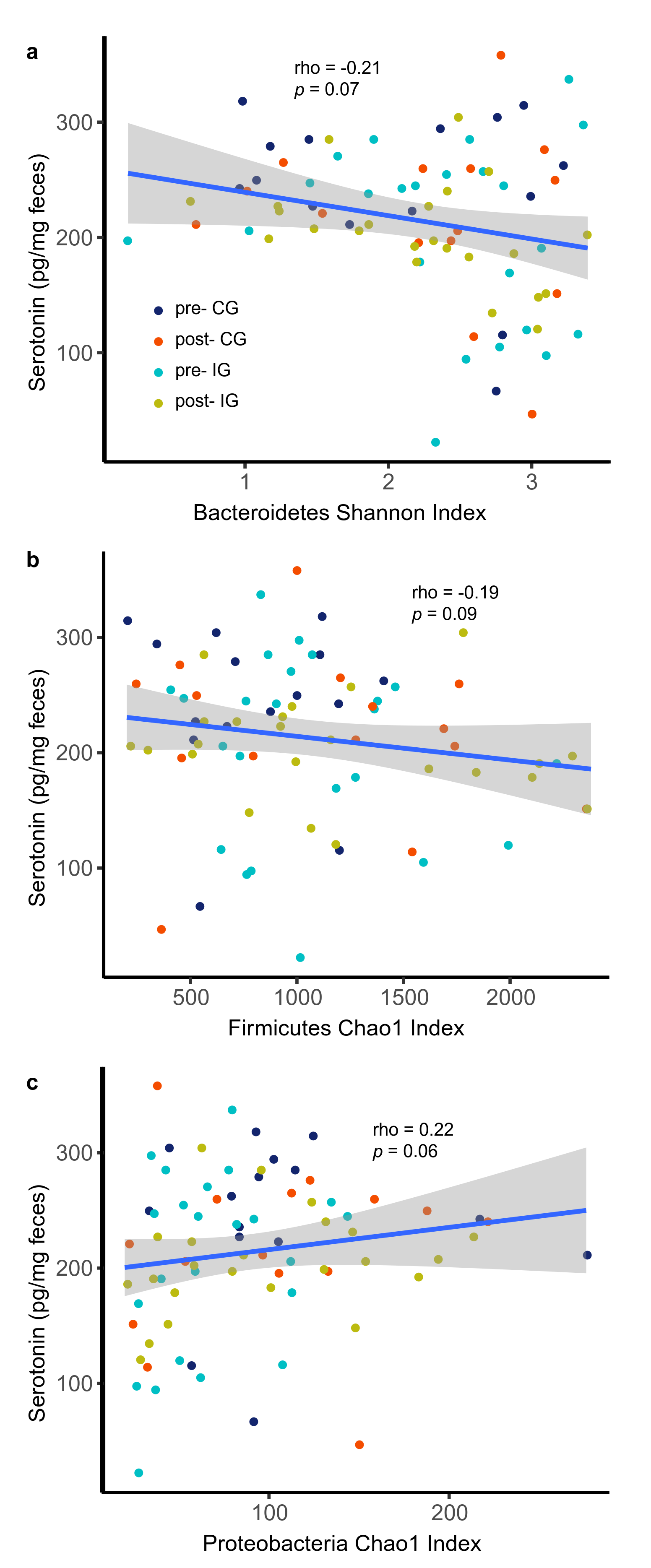


**Supplementary Figure 5.** **Correlation between fecal serotonin and gut microbiota alpha diversity of different phyla.**

a, Correlation between fecal serotonin and Shannon index of Bacteroidetes. b, Correlation between fecal serotonin and Chao1 index of Firmicutes. c, Correlation between fecal serotonin and Chao1 index of Proteobacteria. One node is one sample. Different color of node indicated the different sample group.


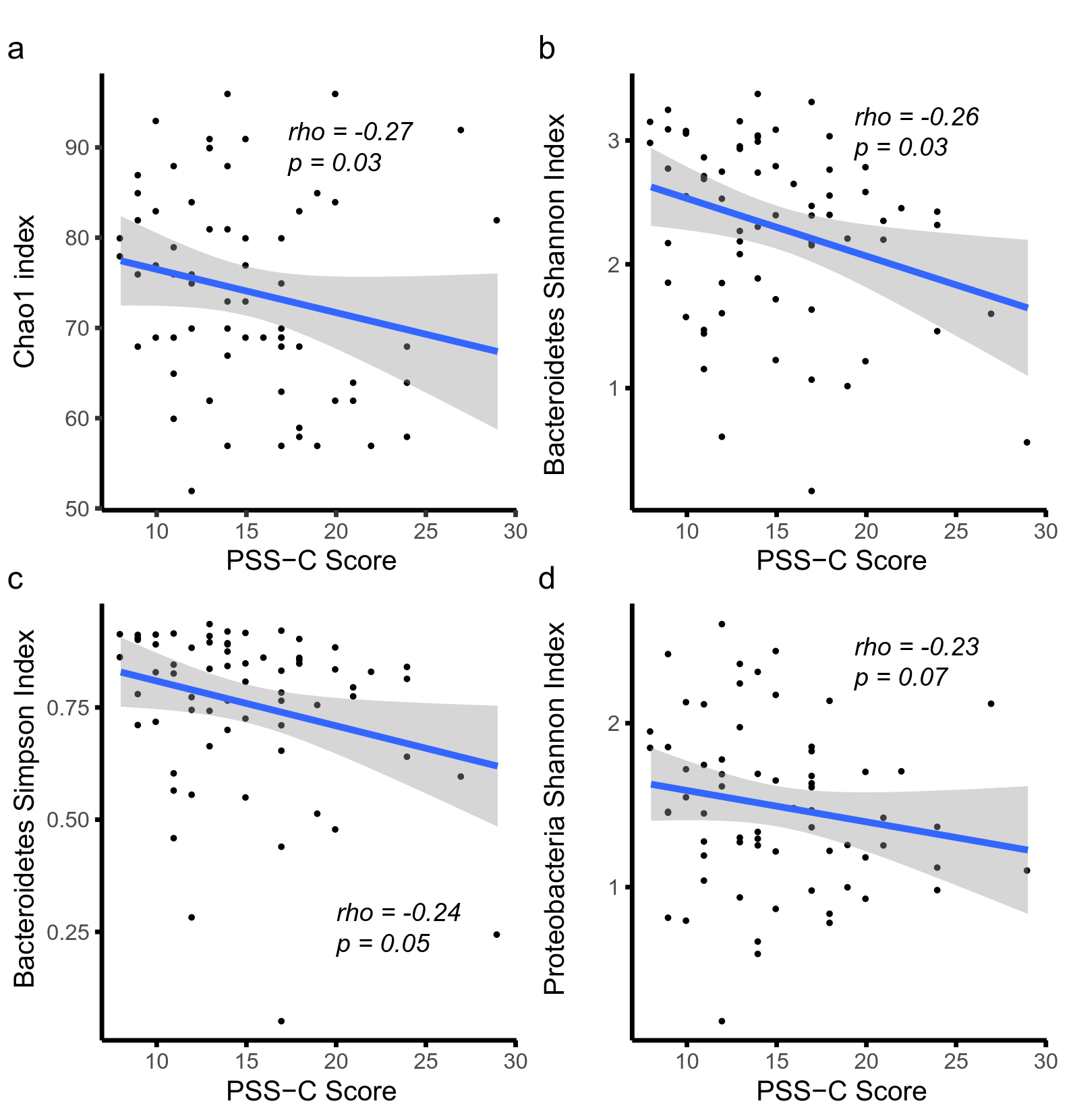


**Supplementary Figure 6.** **Correlation between PSS-C score and gut microbiota alpha diversity.**

a, Correlation between PSS-C score and Chao1 index. b, Correlation between PSS-C score and Shannon index of Bacteroidetes. c, Correlation between PSS-C score and Simpson index of Bacteroidetes. d, Correlation between PSS-C score and Shannon index of Proteobacteria. One node is one sample.


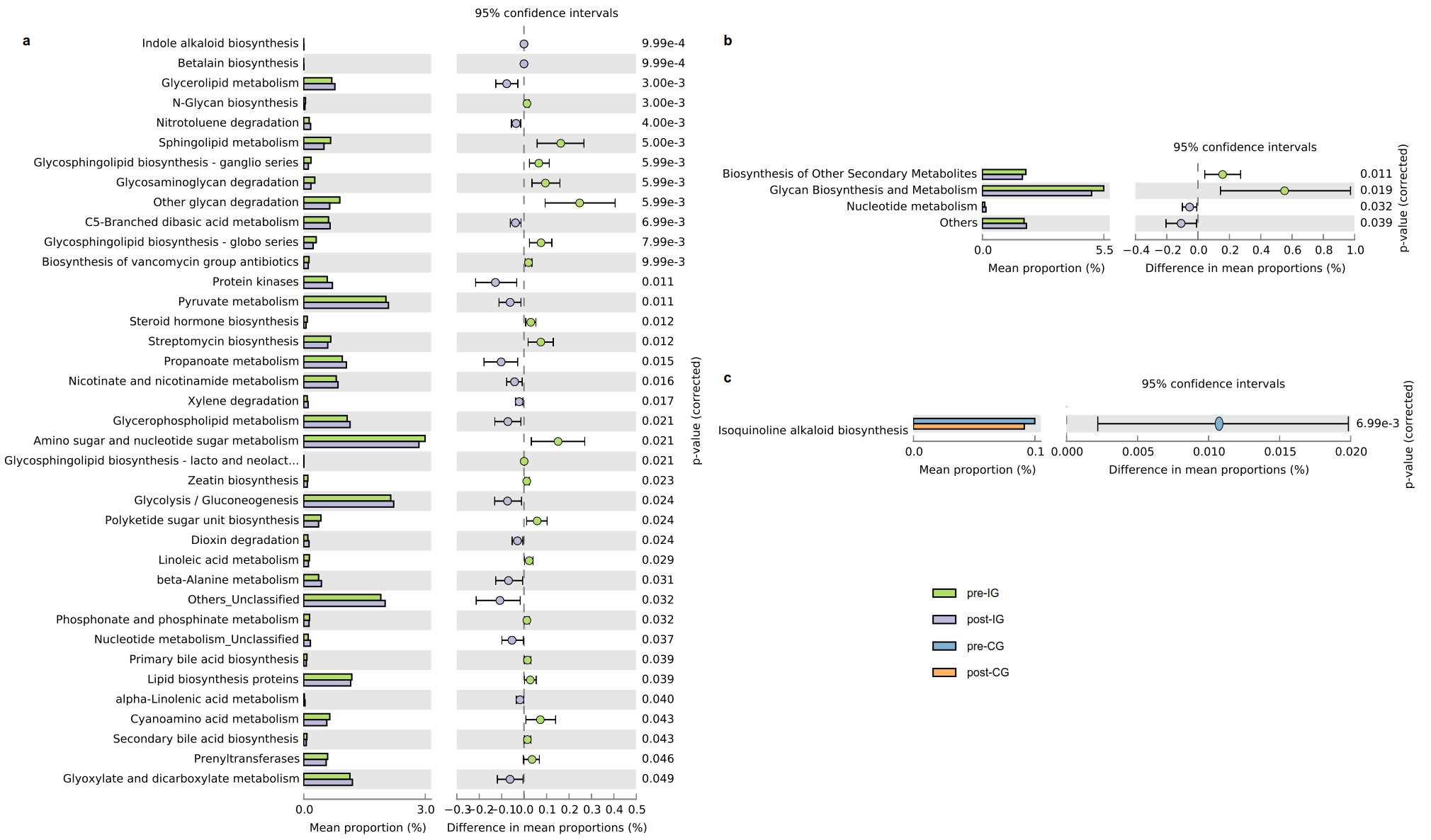


**Supplementary Figure 7.** **Significantly alteration in pathway level based on STAMP.**

a, Significantly alteration level 3 pathway in IG group. b, Significantly alteration level 2 pathway in IG group. c, Significantly alteration level 3 pathway in CG group.

| **Supplemental Table** | | | |  |  |  |  |
| --- | --- | --- | --- | --- | --- | --- | --- |
| **Supplementary Table 1. Participant demographics** | | | | |  |  |  |
|  | | | | **Intervention (n = 27)  n (%) or mean (SD)** | **Control (n = 18)  n (%) or mean (SD)** | **t / χ2** | **p** |
|  |  |  |  |  |  |  |  |
| **Children** | | | |  | | | |
|  | Age (month) | |  | 35.8 (12.21) | 35.4 (8.51) | 0.13 | 0.898 |
|  | Gender | | *Boys* | 13 (48.15) | 10 (55.56) | 0.24 | 0.626 |
|  | Birth weight (g) | |  | 3305.6 (984.41) | 3059.0 (294.54) | 0.88 | 0.387 |
|  | Birth length (cm) | |  | 51.3 (3.51) | 49.6 (3.98) | 1.12 | 0.274 |
|  | Sleeping time at night | |  | 9:30 PM (1:20) | 9:48 PM (0:57) | -0.89 | 0.378 |
|  | Sleep duration per night (hours) | | | 10.0 (0.73) | 9.7 (0.69) | 1.53 | 0.133 |
|  | Daily vegetable intake | *1 bowl or above* | | 14 (51.85) | 8 (44.44) | 5.13 | 0.275 |
|  | Daily fruit intake | *1 medium-sized or above* | | 18 (66.67) | 10 (55.56) | 2.84 | 0.585 |
| **Families** | | | |  | | | |
|  | Mother's age (year) | |  | 37.3 (4.39) | 38.1 (4.40) | 0.55 | 0.584 |
|  | Father's nationality | | *Chinese* | 18 (66.67) | 14 (77.78) | 0.39 | 0.531 |
|  | Mother's nationality | | *Chinese* | 21 (77.78) | 15 (83.33) | 0.05 | 0.828 |
|  | Family structure | | *Nuclear family* | 18 (66.67) | 15 (83.33) | 0.75 | 0.385 |
|  | Family monthly income | | *> 40000 HKD* | 20 (74.07) | 12 (66.67) | 0.30 | 0.862 |
|  | Father’s education level | | *Post-secondary or above* | 25 (92.59) | 16 (88.89) | 2.85 | 0.240 |
|  | Mother’s education level | | *Post-secondary or above* | 26 (96.30) | 17 (94.44) | 0.09 | 0.768 |
|  | Father’s work situation | | *Employed* | 22 (81.48) | 13 (72.22) | 1.00 | 0.316 |
|  | Mother’s work situation | | *Employed* | 10 (37.04) | 7 (38.89) | 2.35 | 0.671 |

|  | | | |  |  |  |  |
| --- | --- | --- | --- | --- | --- | --- | --- |
| **Supplementary Table 1a. Demographics of intervention group (IG) and its dropout at baseline** | | | | | | | |
|  | | | | **Intervention (n = 27)  n (%) or mean (SD)** | **Dropout in IG (n = 3)  n (%) or mean (SD)** | **t / χ2** | **p** |
|  |  |  |  |  |  |  |  |
| **Children** | | | |  | | | |
|  | Age (month) | |  | 35.8 (12.21) | 28.3 (6.66) | 1.03 | 0.311 |
|  | Gender | | *Boys* | 13 (48.15) | 0 (0.00) | 2.55 | 0.110 |
|  | Birth weight (g) | |  | 3305.6 (984.41) | 3118.3 (547.18) | 0.32 | 0.755 |
|  | Birth length (cm) | |  | 51.3 (3.51) | 52.3 (6.66) | -0.39 | 0.702 |
|  | Sleeping time at night | |  | 9:30 PM (1:20) | 9:30 PM (0:30) | 1.10 | 0.312 |
|  | Sleep duration per night (hours) | | | 10.0 (0.73) | 9.3 (0.58) | 1.51 | 0.141 |
|  | Daily vegetable intake | *1 bowl or above* | | 14 (51.85) | 0 (0) | 6.26 | 0.100 |
|  | Daily fruit intake | *1 medium-sized or above* | | 18 (66.67) | 1 (33.33) | 3.56 | 0.465 |
| **Families** | | | |  | | | |
|  | Mother's age (year) | |  | 37.3 (4.39) | 35.0 (3.00) | 0.89 | 0.381 |
|  | Father's nationality | | *Chinese* | 18 (66.67) | 3 (100) | 1.28 | 0.259 |
|  | Mother's nationality | | *Chinese* | 21 (77.78) | 3 (100) | 0.70 | 0.404 |
|  | Family structure | | *Nuclear family* | 18 (66.67) | 1 (33.33) | 1.84 | 0.175 |
|  | Family monthly income | | *> 40000 HKD* | 20 (74.07) | 3 (100) | 3.12 | 0.210 |
|  | Father’s education level | | *Post-secondary or above* | 25 (92.59) | 3 (100) | 0.25 | 0.619 |
|  | Mother’s education level | | *Post-secondary or above* | 26 (96.30) | 3 (100) | 0.12 | 0.730 |
|  | Father’s work situation | | *Employed* | 22 (81.48) | 2 (66.67) | 0.55 | 0.459 |
|  | Mother’s work situation | | *Employed* | 10 (37.04) | 3 (100) | 4.12 | 0.390 |

|  | | | |  |  |  |  |
| --- | --- | --- | --- | --- | --- | --- | --- |
| **Supplementary Table 1b. Demographics of control group (CG) and its dropout at baseline** | | | | | | | |
|  | | | | **Control (n = 18)  n (%) or mean (SD)** | **Dropout in CG (n = 6)  n (%) or mean (SD)** | **t / χ2** | **p** |
|  |  |  |  |  |  |  |  |
| **Children** | | | |  | | | |
|  | Age (month) | |  | 35.4 (8.51) | 36.4 (6.19) | -0.25 | 0.808 |
|  | Gender | | *Boys* | 10 (55.56) | 3 (50.00) | 0.06 | 0.813 |
|  | Birth weight (g) | |  | 3059.0 (294.54) | 2725.8 (597.10) | 1.30 | 0.241 |
|  | Birth length (cm) | |  | 49.6 (3.98) | 49.7 (4.97) | -0.04 | 0.967 |
|  | Sleeping time at night | |  | 9:48 PM (0:57) | 9:35 PM (1:04) | 0.48 | 0.636 |
|  | Sleep duration per night (hours) | | | 10.0 (0.73) | 10.2 (0.75) | -1.51 | 0.145 |
|  | Daily vegetable intake | *1 bowl or above* | | 8 (44.44) | 5 (83.33) | 6.23 | 0.157 |
|  | Daily fruit intake | *1 medium-sized or above* | | 10 (55.56) | 4 (66.67) | 1.76 | 0.623 |
| **Families** | | | |  | | | |
|  | Mother's age (year) | |  | 38.1 (4.40) | 34.8 (3.54) | 1.65 | 0.114 |
|  | Father's nationality | | *Chinese* | 14 (77.78) | 5 (83.33) | 0.08 | 0.772 |
|  | Mother's nationality | | *Chinese* | 15 (83.33) | 5 (83.33) | 1.00 | 1.000 |
|  | Family structure | | *Nuclear family* | 15 (83.33) | 3 (50.00) | 2.67 | 0.102 |
|  | Family monthly income | | *> 40000 HKD* | 12 (66.67) | 5 (83.33) | 1.75 | 0.416 |
|  | Father’s education level | | *Post-secondary or above* | 16 (88.89) | 4 (66.67) | 3.81 | 0.149 |
|  | Mother’s education level | | *Post-secondary or above* | 17 (94.44) | 5 (83.33) | 0.73 | 0.394 |
|  | Father’s work situation | | *Employed* | 13 (72.22) | 4 (66.67) | 0.07 | 0.759 |
|  | Mother’s work situation | | *Employed* | 7 (38.89) | 5 (83.33) | 3.78 | 0.286 |

**Supplementary Table 2. Odds Ratios for anger and adequate sleep frequency in Relation to serotonin.**

|  | **OR (95%CI)** | **P value** |
| --- | --- | --- |
|  | **Anger Frequency (below vs. above 2)** | |
| **Serotonin (below vs above median)** |  |  |
| Crude Model | 0.35 (0.12-0.97) | 0.048 |
| Adjusted Model^1^ | 0.32 (0.10-0.93) | 0.041 |
|  | **Adequate Sleep Frequency (below vs. above 2)** | |
| **Serotonin (below vs above median)** |  |  |
| Crude Model | 6.43 (1.01-125.47) | 0.049 |
| Adjusted Model^1^ | 7.03 (1.06-140.17) | 0.045 |
| ^1^Adjusted for age |  |  |

**Supplementary Table 3. Sequencing data summary of each sample.**

|  | **#Total sequence reads** | **#Trim remain reads** | **#Remain single reads** | **#Remain Pair reads** | **#Match pair reads in Flash** | **#Sequence after Flash** | **#Remove chimera remain reads** | **%Remove chimera remain reads** |
| --- | --- | --- | --- | --- | --- | --- | --- | --- |
| FC-10-1 | 229414 | 186943 | 21619 | 82662 | 5844 | 76818 | 53784 | 70.00% |
| FC-10-2 | 188326 | 157432 | 15900 | 70766 | 6837 | 63929 | 43509 | 68.10% |
| FC-1-1 | 249520 | 210550 | 18734 | 95908 | 6445 | 89463 | 52263 | 58.40% |
| FC-11-1 | 365678 | 311629 | 28361 | 141634 | 7085 | 134549 | 69914 | 52.00% |
| FC-11-2 | 334220 | 284344 | 26616 | 128864 | 6041 | 122823 | 65803 | 53.60% |
| FC-1-2 | 168030 | 135435 | 15843 | 59796 | 5932 | 53864 | 37818 | 70.20% |
| FC-13-1 | 205560 | 176191 | 13433 | 81379 | 4125 | 77254 | 45287 | 58.60% |
| FC-13-2 | 280180 | 238053 | 20715 | 108669 | 4007 | 104662 | 57532 | 55.00% |
| FC-14-1 | 221450 | 189755 | 14467 | 87644 | 5064 | 82580 | 47422 | 57.40% |
| FC-14-2 | 257072 | 219638 | 18028 | 100805 | 5639 | 95166 | 53027 | 55.70% |
| FC-15-1 | 181858 | 151583 | 13135 | 69224 | 5344 | 63880 | 39325 | 61.60% |
| FC-15-2 | 227308 | 194255 | 16043 | 89106 | 4714 | 84392 | 42921 | 50.90% |
| FC-16-1 | 183706 | 153311 | 18885 | 67213 | 2210 | 65003 | 51067 | 78.60% |
| FC-16-2 | 244890 | 209253 | 19429 | 94912 | 3193 | 91719 | 59698 | 65.10% |
| FC-17-1 | 209020 | 175371 | 16983 | 79194 | 5423 | 73771 | 42659 | 57.80% |
| FC-17-2 | 229092 | 192567 | 20001 | 86283 | 5827 | 80456 | 43525 | 54.10% |
| FC-18-1 | 216778 | 182206 | 17000 | 82603 | 5068 | 77535 | 46398 | 59.80% |
| FC-18-2 | 188296 | 156967 | 16239 | 70364 | 4606 | 65758 | 40008 | 60.80% |
| FC-19-1 | 256082 | 214819 | 19197 | 97811 | 7575 | 90236 | 44988 | 49.90% |
| FC-19-2 | 202206 | 171381 | 16097 | 77642 | 5732 | 71910 | 44399 | 61.70% |
| FC-2-1 | 198782 | 169815 | 13051 | 78382 | 6863 | 71519 | 43897 | 61.40% |
| FC-2-2 | 165390 | 138651 | 12097 | 63277 | 4638 | 58639 | 42365 | 72.20% |
| FC-3-1 | 203976 | 174727 | 11009 | 81859 | 6357 | 75502 | 45899 | 60.80% |
| FC-3-2 | 182954 | 159863 | 10037 | 74913 | 3933 | 70980 | 40557 | 57.10% |
| FC-4-1 | 168404 | 144929 | 12823 | 66053 | 2606 | 63447 | 48256 | 76.10% |
| FC-4-2 | 223350 | 177488 | 32486 | 72501 | 2088 | 70413 | 45023 | 63.90% |
| FC-5-1 | 219878 | 177464 | 29572 | 73946 | 2128 | 71818 | 48885 | 68.10% |
| FC-5-2 | 185174 | 145977 | 26171 | 59903 | 2313 | 57590 | 39782 | 69.10% |
| FC-6-1 | 254158 | 217945 | 17683 | 100131 | 7332 | 92799 | 56189 | 60.50% |
| FC-6-2 | 231746 | 194614 | 16170 | 89222 | 7455 | 81767 | 55404 | 67.80% |
| FC-7-1 | 261146 | 225820 | 17060 | 104380 | 3931 | 100449 | 51475 | 51.20% |
| FC-7-2 | 316614 | 273788 | 19996 | 126896 | 4352 | 122544 | 65686 | 53.60% |
| FC-8-1 | 293904 | 248173 | 23249 | 112462 | 7193 | 105269 | 60529 | 57.50% |
| FC-8-2 | 313614 | 264454 | 27686 | 118384 | 5451 | 112933 | 59891 | 53.00% |
| FC-9-1 | 221990 | 186900 | 18120 | 84390 | 4039 | 80351 | 48201 | 60.00% |
| FC-9-2 | 316656 | 269140 | 24692 | 122224 | 4818 | 117406 | 62635 | 53.30% |
| FI-10-1 | 209722 | 174118 | 18634 | 77742 | 4199 | 73543 | 43689 | 59.40% |
| FI-10-2 | 214418 | 176501 | 21619 | 77441 | 4106 | 73335 | 46401 | 63.30% |
| FI-1-1 | 327822 | 243079 | 72213 | 85433 | 2443 | 82990 | 49898 | 60.10% |
| FI-11-1 | 114316 | 96776 | 8670 | 44053 | 1642 | 42411 | 33453 | 78.90% |
| FI-11-2 | 112050 | 95320 | 9826 | 42747 | 1560 | 41187 | 31173 | 75.70% |
| FI-1-2 | 431084 | 313175 | 102595 | 105290 | 1803 | 103487 | 56386 | 54.50% |
| FI-12-1 | 166274 | 139249 | 11671 | 63789 | 5373 | 58416 | 38634 | 66.10% |
| FI-12-2 | 174866 | 146040 | 17756 | 64142 | 3166 | 60976 | 39775 | 65.20% |
| FI-13-1 | 186712 | 150747 | 22103 | 64322 | 3494 | 60828 | 37118 | 61.00% |
| FI-13-2 | 201792 | 165932 | 23166 | 71383 | 2582 | 68801 | 41399 | 60.20% |
| FI-14-1 | 173418 | 140120 | 22254 | 58933 | 2463 | 56470 | 38485 | 68.20% |
| FI-14-2 | 180098 | 153608 | 11530 | 71039 | 4807 | 66232 | 46617 | 70.40% |
| FI-15-1 | 139142 | 118946 | 9236 | 54855 | 3020 | 51835 | 33145 | 63.90% |
| FI-15-2 | 167986 | 141910 | 10064 | 65923 | 6551 | 59372 | 39757 | 67.00% |
| FI-16-1 | 256898 | 219446 | 14796 | 102325 | 7909 | 94416 | 53625 | 56.80% |
| FI-16-2 | 307364 | 263514 | 22078 | 120718 | 5384 | 115334 | 59370 | 51.50% |
| FI-18-1 | 229526 | 190041 | 16143 | 86949 | 6490 | 80459 | 47958 | 59.60% |
| FI-18-2 | 144634 | 118984 | 11918 | 53533 | 2176 | 51357 | 35493 | 69.10% |
| FI-19-1 | 240214 | 201828 | 19438 | 91195 | 4411 | 86784 | 46336 | 53.40% |
| FI-19-2 | 199878 | 175416 | 9896 | 82760 | 3338 | 79422 | 45487 | 57.30% |
| FI-20-1 | 173112 | 151820 | 8530 | 71645 | 3778 | 67867 | 40135 | 59.10% |
| FI-20-2 | 211388 | 180682 | 12086 | 84298 | 6773 | 77525 | 51295 | 66.20% |
| FI-2-1 | 321926 | 272016 | 30292 | 120862 | 4208 | 116654 | 71786 | 61.50% |
| FI-21-1 | 184996 | 161444 | 9086 | 76179 | 4386 | 71793 | 43228 | 60.20% |
| FI-21-2 | 224158 | 195405 | 12749 | 91328 | 5011 | 86317 | 47675 | 55.20% |
| FI-2-2 | 387880 | 329619 | 35531 | 147044 | 3544 | 143500 | 74073 | 51.60% |
| FI-22-1 | 333148 | 281820 | 22278 | 129771 | 5006 | 124765 | 59016 | 47.30% |
| FI-22-2 | 286984 | 246434 | 17770 | 114332 | 3789 | 110543 | 56362 | 51.00% |
| FI-23-1 | 209604 | 178813 | 14859 | 81977 | 5221 | 76756 | 43345 | 56.50% |
| FI-23-2 | 172968 | 147440 | 10760 | 68340 | 3697 | 64643 | 44911 | 69.50% |
| FI-24-1 | 252768 | 221226 | 15012 | 103107 | 3731 | 99376 | 52768 | 53.10% |
| FI-24-2 | 216550 | 186264 | 12370 | 86947 | 6248 | 80699 | 51282 | 63.50% |
| FI-25-1 | 161694 | 136488 | 11108 | 62690 | 4153 | 58537 | 40220 | 68.70% |
| FI-25-2 | 189870 | 161548 | 14730 | 73409 | 4247 | 69162 | 44199 | 63.90% |
| FI-26-1 | 321240 | 266172 | 28580 | 118796 | 8564 | 110232 | 59442 | 53.90% |
| FI-26-2 | 284992 | 231971 | 37603 | 97184 | 4840 | 92344 | 50792 | 55.00% |
| FI-27-1 | 246902 | 211561 | 16249 | 97656 | 6810 | 90846 | 52695 | 58.00% |
| FI-27-2 | 254448 | 218857 | 16331 | 101263 | 5061 | 96202 | 52780 | 54.90% |
| FI-29-1 | 275364 | 234738 | 16924 | 108907 | 7273 | 101634 | 56652 | 55.70% |
| FI-29-2 | 205406 | 178174 | 14180 | 81997 | 2701 | 79296 | 49184 | 62.00% |
| FI-30-1 | 286694 | 250519 | 15809 | 117355 | 3551 | 113804 | 60274 | 53.00% |
| FI-30-2 | 232518 | 198502 | 16222 | 91140 | 4237 | 86903 | 48614 | 55.90% |
| FI-3-1 | 372502 | 309743 | 34549 | 137597 | 6991 | 130606 | 73066 | 55.90% |
| FI-3-2 | 282834 | 236678 | 25418 | 105630 | 4868 | 100762 | 60172 | 59.70% |
| FI-4-1 | 306948 | 241279 | 52143 | 94568 | 3466 | 91102 | 52829 | 58.00% |
| FI-4-2 | 450950 | 333325 | 95219 | 119053 | 2859 | 116194 | 62309 | 53.60% |
| FI-5-1 | 434912 | 339744 | 75346 | 132199 | 3190 | 129009 | 70763 | 54.90% |
| FI-5-2 | 385000 | 285370 | 84450 | 100460 | 1723 | 98737 | 53400 | 54.10% |
| FI-6-1 | 158182 | 134444 | 10494 | 61975 | 3678 | 58297 | 41491 | 71.20% |
| FI-6-2 | 224680 | 192619 | 14937 | 88841 | 5020 | 83821 | 51927 | 61.90% |
| FI-8-1 | 222546 | 185321 | 16363 | 84479 | 5546 | 78933 | 53185 | 67.40% |
| FI-8-2 | 207958 | 174293 | 14799 | 79747 | 5554 | 74193 | 48591 | 65.50% |
| FI-9-1 | 262686 | 222251 | 20563 | 100844 | 6149 | 94695 | 50636 | 53.50% |
| FI-9-2 | 105082 | 83674 | 10986 | 36344 | 2877 | 33467 | 32664 | 97.60% |

# indicated the number of reads in each step of the filter process. % indicated the percentage of reads.

**Supplementary Table 4. Odds ratios for serotonin in relation to gut microbiota measurements.**

| **Microbiota Measurements** | **Crude Model** | | **Adjusted Model^1^** | |
| --- | --- | --- | --- | --- |
|  | **OR (95% CI)** | **P value** | **OR (95% CI)** | **P value** |
| Eggerthella_lenta | 1.70 (0.69-4.26) | 0.253 | 1.75 (0.68-4.61) | 0.251 |
| Bacteroides_eggerthii | 0.31 (0.09-0.95) | 0.048 | 0.39 (0.11-1.22) | 0.115 |
| g__Porphyromonas_species_unclassify | 0.17 (0.02-0.69) | 0.027 | 0.15 (0.02-0.64) | 0.021 |
| f__Rikenellaceae_species_unclassify | 0.26 (0.10-0.67) | 0.006 | 0.29 (0.10-0.75) | 0.013 |
| o__Streptophyta_species_unclassify | 0.18 (0.06-0.49) | 0.001 | 0.17 (0.05-0.50) | 0.002 |
| Staphylococcus_aureus | 3.86 (1.04-18.62) | 0.058 | 3.17 (0.79-16.13) | 0.123 |
| g__Lactobacillus_species_unclassify | 0.18 (0.04-0.62) | 0.012 | 0.19 (0.04-0.68) | 0.018 |
| Streptococcus_anginosus | 3.86 (1.04-18.62) | 0.058 | 4.26 (1.11-21.35) | 0.048 |
| f__Lachnospiraceae_Other | 0.47 (0.19-1.17) | 0.110 | 0.43 (0.16-1.14) | 0.094 |
| f__Lachnospiraceae_species_unclassify | 0.47 (0.19-1.17) | 0.110 | 0.43 (0.15-1.15) | 0.096 |
| g__Roseburia_Other | 0.29 (0.11-0.73) | 0.011 | 0.25 (0.09-0.67) | 0.007 |
| Roseburia_faecis | 0.32 (0.12-0.81) | 0.019 | 0.32 (0.11-0.87) | 0.028 |
| g__WAL_1855D_species_unclassify | 0.35 (0.09-1.17) | 0.105 | 0.34 (0.09-1.16) | 0.099 |
| g__Coprobacillus_species_unclassify | 2.69 (1.05-7.13) | 0.041 | 4.00 (1.39-12.69) | 0.013 |
| f__mitochondria_Other | 0.19 (0.03-0.81) | 0.043 | 0.18 (0.03-0.79) | 0.040 |
| g__Morganella_Other | 1.89 (0.76-4.76) | 0.172 | 2.08 (0.81-5.47) | 0.131 |
| Trabulsiella_farmeri | 4.74 (1.48-18.45) | 0.014 | 5.65 (1.56-27.15) | 0.014 |
| ^1^Adjusted for age |  |  |  |  |
